# Exploratory Spatial Analysis of *in vitro* Respiratory Syncytial Virus Co-infections

**DOI:** 10.3390/v2122782

**Published:** 2010-12-22

**Authors:** Ivan Simeonov, Xiaoyan Gong, Oekyung Kim, Mary Poss, Francesca Chiaromonte, John Fricks

**Affiliations:** 1 Department of Statistics, Pennsylvania State University, University Park, PA 16802, USA; 2 Department of Biology, Pennsylvania State University, University Park, PA 16802, USA; 3 Fogarty International Center, National Institutes of Health, Bethesda, MD 20892, USA

**Keywords:** human respiratory syncytial virus, co-infections, spatial statistics

## Abstract

The cell response to virus infection and virus perturbation of that response is dynamic and is reflected by changes in cell susceptibility to infection. In this study, we evaluated the response of human epithelial cells to sequential infections with human respiratory syncytial virus strains A2 and B to determine if a primary infection with one strain will impact the ability of cells to be infected with the second as a function of virus strain and time elapsed between the two exposures. Infected cells were visualized with fluorescent markers, and location of all cells in the tissue culture well were identified using imaging software. We employed tools from spatial statistics to investigate the likelihood of a cell being infected given its proximity to a cell infected with either the homologous or heterologous virus. We used point processes, K-functions, and simulation procedures designed to account for specific features of our data when assessing spatial associations. Our results suggest that intrinsic cell properties increase susceptibility of cells to infection, more so for RSV-B than for RSV-A. Further, we provide evidence that the primary infection can decrease susceptibility of cells to the heterologous challenge virus but only at the 16 h time point evaluated in this study. Our research effort highlights the merits of integrating empirical and statistical approaches to gain greater insight on *in vitro* dynamics of virus-host interactions.

## 1. Introduction

Infections of the respiratory tract account for millions of death annually, exacting the highest toll in infants and small children. Among infections caused by viruses, human respiratory syncytial virus (RSV) comprises up to 70% of cases of children hospitalized with bronchiolitis [1–3]. Approximately two-thirds of infants are infected with RSV during the first year of life, and 90% have been infected one or more times by 2 years of age [4].

RSV is a Paramyxovirus and has been classified into two strains, RSV-A and RSV-B, based on both antigenic and sequence data. Although both strains circulate, typically only one is responsible for each seasonal outbreak and may be the dominant strain for several years before being replaced by the other [5,6]. There is neither protection nor cross protection conferred by infection with these viruses [7]; children can be infected with either strain of RSV twice during seasonal outbreaks [8]. The lack of apparent adaptive immunity and the strong age predilection for disease observed with RSV infection indicate that control of viral infection by innate immunity is important. In addition, RSV is unique among the members of the Paramyxoviridae, because it encodes two genes, NS1 and NS2, that abrogate the interferon response [9,10]. Thus, RSV has made a substantial evolutionary investment in controlling the host innate immune response.

In this study, we explored cell responses to RSV using heterologous infections with RSV-B and RSV-A in an *in vitro* experimental system. We hypothesized that a primary infection with one strain would elicit an innate response over time that would render surrounding cells refractory to a challenge infection with the other strain. The innate response could be elicited by direct contact of cells with infected cells or by diffusion of soluble mediators through the culture media. The experiments were designed to allow a single cycle of infection; infection by the challenge virus occurred prior to release of infectious particles by the primary virus. Briefly, the experimental set up is as follows: Wells plated with a human epithelial cell line are exposed to a primary infection with one strain of the virus and, after a time lag, are challenged with the second strain of the virus. Fluorescent stains allow us to visualize cell nuclei along with RSV-B and RSV-A infected cells in each well. Image analysis software is then used to produce aggregate counts and two dimensional spatial coordinates for nuclei and infection “marks”.

Aggregate counts can be used to glean some features of infections and co-infections. However, richer information can be obtained analyzing the spatial structure of infections within each well. A spatial analysis allows us to characterize the infection status of cells as a function of their location and proximity to one another. In turn, this allows us to explore how susceptibility may be affected by local conditions interacting with cell properties or producing innate immune responses. In particular, we are interested in detecting spatial association in the form of attraction or repulsion among cells infected with the same or different strains of the virus (roughly speaking, the number of infected cells surrounding any given infected cell on average). Attraction and repulsion capture the tendency of cells surrounding an infected cell to have higher or lower susceptibility to infection; a significant attraction suggests intrinsic cell properties or innate responses lead to increased susceptibility. Conversely, a significant repulsion suggests changes leading to decreased susceptibility.

In Section 2, we introduce the main ingredients in such an analysis. We discuss the concept and basic definitions of spatial point processes. In addition, we introduce *K*-functions, which capture spatial association within one or between two spatial point processes on a range of scales, along with standard methodology to estimate such functions. While aggregate counts of infection marks are satisfactory proxies for the number of infected cells in a well, features of our experimental system (including the nature of the fluorescent dyes used to visualize nuclei and infections) and limits in the image analysis software render the two dimensional mark coordinates at our disposal imperfect proxies for the locations of cells and infected cells. In Section 2, we also describe simulation procedures that we designed to detect significant spatial association while accounting for these issues. Detecting such an association provides evidence for local effects of susceptibility to infection.

## 2. Statistical Framework

**Figure 1:**
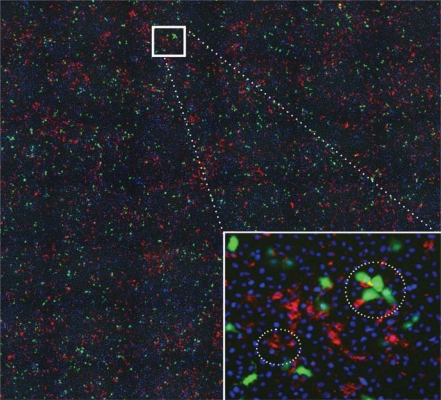
An example well image presenting visual evidence of “clumping” among infected cells. Blue stains represent cell nuclei, red stains are located on the membrane of cells infected with RSV-B, and green stains are located in the cytoplasm of cells infected with GFP-RSV-A. The inset in the bottom right corner enlarges a region of the well, showing the stains in detail. In the small circled region, we observe how several B stains can lay in close proximity to one another and to more than one nucleus. This proximity could indicate neighboring cells infected with RSV-B or multiple staining on the membrane of the same infected cell. In the large circled region we observe how A stains “fill” the volume of cells, sometimes blurring into one another. We also note very close B and A stains, which could indicate two neighboring cells infected with the two strains or which may be due to a double infection of the same cell (again large circle in the inset).

In the imaging of the cell cultures, the location and infection status of cells are visualized through different types of staining techniques. Cell nuclei are rendered through DAPI stains. RSV-A infected cells are rendered through cytoplasmic stains (GFP-RSVA2 is detected by expression of GFP in cell cytoplasm), and RSV-B infected cells are rendered through membrane stains (using Alexa fluor 568 labeled antibody to viral F protein). For illustration, the inset on the right of Figure 1 shows stains in a selected region of one well image.

A natural statistical framework for these data are spatial point processes in the plane; in particular, the two dimensional coordinates of the centers of the stains detected by the imaging software of nuclei, A marks, and B marks in any given well can be thought of as realizations of three such processes. A spatial point process models a random collection of discrete points in the plane. A common example is the Poisson random field, which we will use as a baseline for comparison. In a Poisson random field, **X**, the number of points in any reasonable subset of the plane has a Poisson distribution with mean equal to a constant, λ, times the area of the subset. The constant λ is called the intensity and represents the expected number of points per unit area. Another defining property of the Poisson random field is that the number of points in any two non-overlapping subsets is independent. This implies that a point pattern generated by a Poisson random field, say **x** = (*x*_1_, …, *x*_*n*_**x**__), where *n***_x_** is the number of points in the pattern, is uniformly distributed across the observation area with no attraction or repulsion among the points. Each point is “ignorant” of the other points in the data.

For a Poisson random field, as well as a generic spatial point process, assuming that the mean number of points in any subset is proportional to the area and does not depend on the particular location of the subset is referred to as *homogeneity*. In some applications, one may need to relax this assumption and use an *inhomogeneous* processes to model the data. For such processes, intensity is a non-constant function in the plane, which could be written as λ(*x*), and the average number of points in a subset *A* would be ∫*_A_* λ(*x*)*dx*. For the work presented here, we checked for homogeneity with statistical tests and determined that we do not need to resort to inhomogeneous processes to model our nuclei and infection marks. This is important, because the latter can be more complicated to analyze (see Section 5). Inhomogeneous point processes will be mentioned again when discussing related work in Section 4.

An important descriptor of a spatial point process **X** is Ripley’s *K*-function, which measures the tendency of points generated by the process to attract or repel one another at various ranges. For a homogeneous process, the *K*-function is defined as [11]

$$\begin{array}{l} K(r)=\frac{1}{\left|A\right|}E\left[\sum_{x\in \text{X}\cap A}\frac{1}{\lambda }\sum_{\tilde{x}\in \text{X},\tilde{x}\neq x}\frac{1_{\left\{d(x,\tilde{x})\leq r\right\}}}{\lambda }\right]\\ \;\;=\frac{1}{\left|A\right|\lambda^{2}}E\left[\sum_{x\in \text{X}\cap A}\sum_{\tilde{x}\in \text{X},\tilde{x}\neq x}1_{\left\{d(x,\tilde{x})\leq r\right\}}\right] \end{array}$$

where *A* is any subset, *E* [·] is an expected value, 1_{·}_ is an indicator function of an event, *d*(·, ·) is a distance between two points, and *r* is a radius. Intuitively, *K*(*r*) captures the spatial accumulation of points in neighborhoods of increasing radius. More precisely, λ*K*(*r*) is the expected number of points in a circle of radius *r* around a “typical” point of the process. For a homogeneous Poisson random field, the value of *K*(*r*) is π*r*^2^ [11]. Thus, when there is no attraction or repulsion, the expected number of points around a “typical” point increases proportionally to the square of the radius, *i.e.*, proportionally to the area of the circle under consideration. Note that this descriptor is well-defined only if the spatial point process is stationary [11]. It is also important to note that the terms ”attraction” and ”repulsion” as they are used here adhere to their typical use in the spatial point processes literature. The term “attraction” refers to an increased propensity to accumulate points near a typical point, and “repulsion” to the opposite. This does not mean that infected cells physically attract or repel one another in space, but rather that cells tend to share (attraction) or oppose (repulsion) the infection status of neighboring cells in the culture.

A similar descriptor can be formulated when considering two point processes, say **X** and **Y**, to measure the tendency of points generated by one process to repel or attract points generated by the other. This is called the the cross *K*-function; for two homogeneous processes, it is given by [11]

$$\begin{array}{l} K_{\text{X},\text{Y}}(r)=\frac{1}{\left|A\right|}E\left[\sum_{x\in \text{X}\cap A}\frac{1}{\lambda_{\text{X}}}\sum_{y\in \text{Y}}\frac{1_{\left\{d(x,y)\leq r\right\}}}{\lambda_{\text{Y}}}\right]\\ \;\;\;=\frac{1}{\left|A\right|\lambda_{\text{X}}\lambda_{\text{Y}}}E\left[\sum_{x\in \text{X}\cap A}\sum_{y\in \text{Y}}1_{\left\{d(x,y)\leq r\right\}}\right]. \end{array}$$

Note that the cross *K*-function is symmetric relative to the two processes, *i.e.*, *K*_**X**,**Y**_(*r*) = *K*_**Y**,**X**_(*r*). Moreover, if the two processes are independent, we have that *K*_**X**,**Y**_(*r*) = π*r*^2^, regardless of whether they are Poisson random fields. For the cross *K*-function, quadratic growth is indicative of lack of attraction or repulsion between the points of the two processes [11].

Next, we briefly describe how *K*-functions can be estimated. Suppose we have a point pattern **x** = (*x*_1_, …, *x*_*n*_**x**__) generated by a process **X**, which we assume is homogeneous on an observation window *W*. Estimating its *K*-function requires: (i) estimating the (constant) intensity, (ii) selecting an appropriate region *W_R_* within *W* to perform the calculation, and (iii) counting the number of observed points within a radius *r* from each observed point in *W_R_*. Under homogeneity, an unbiased estimate of the intensity is $\hat{\lambda }=\frac{n_{\text{x}}}{\left|W\right|}$, where *|W|* denotes the area of *W*. This is also the maximum likelihood estimate in the case of a Poisson field [11]. Since we lack knowledge about the process outside *W*, we necessarily underestimate the number of neighbors when considering points close to the boundary of *W*. This bias is often referred to as an “edge effect”. If the data at our disposal is fairly large, we may remedy this issue by discarding some of the data and restricting attention to points within a smaller region *W_R_* removed from the boundary of *W*. Assuming we want to estimate the *K*-function on a range of radii from 0 to *R*, we define *W_R_* by eliminating a “buffer” of width *R* inward from the boundary. In this fashion, we will be able to observe and count all neighbors about each observed point *x_i_* ∈ *W_R_* for any *r* ≤ *R*. Our formula for the estimation of the *K*-function is

$$\hat{K}(r)=\frac{1}{\left|W_{R}\right|{\hat{\lambda }}^{2}}\sum_{i:x_{i}\in W_{R}}\sum_{j\neq i}1_{\left\{d(x_{i},x_{j})\leq r\right\}}.$$

**Figure 2:**
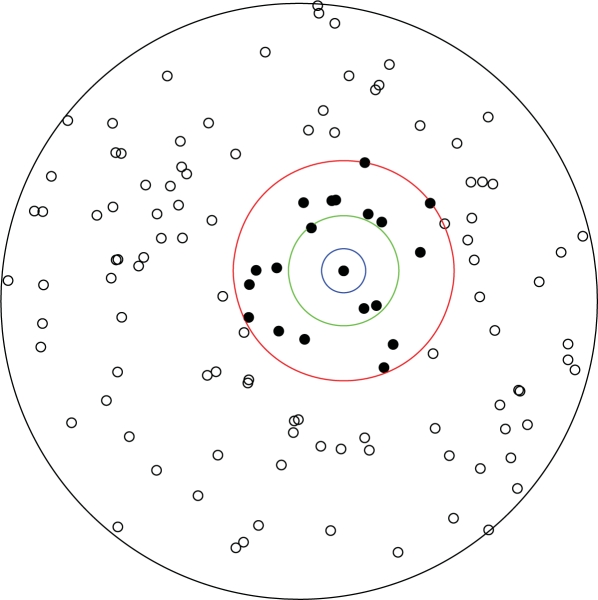
A point pattern generated by a homogeneous Poisson random field (λ = 10^−5^) on a circular observation window of radius 1350. The blue, green and red circles illustrate neighborhoods of increasing radius (*r* = 100, 250, 500) around a given point in the pattern. Counts of points within such neighborhoods (here 0, 3, 19 excluding the center point itself) form the basis for estimation of the *K*-function.

In order to develop some intuition on the estimation of the *K*-function, it is useful to focus on Figure 2. Looking at the estimation formula we notice that the inner summation counts how many points are within a circle of radius *r* from a given point *x_i_*. For illustrative purposes, consider the three circles shown in Figure 2 and assume that the point in the center of these three circles is the point *x_i_*.

For the blue circle with radius *r* of 100, this count is zero, since we do not have any other points within its boundaries. If we repeat this counting for each point (*i.e.*, the outer summation in the estimation formula) and then divide by |*W_R_*|λ̂^2^, we produce an estimate of the *K*-function for *r* equal to 100. Next, the green circle represents a circle with a radius *r* equal to 250. There are 3 different points within the green circle’s boundaries (inner sum). If we place a green circle around each of the points in the point pattern, count the number of different points (inner sum), add those contributions (outer sum), and then divide again by |*W_R_*|λ̂^2^, we produce the estimated *K*-function for *r* = 250. Next is the red circle, which has a radius *r* of 500, and we see that there are a greater number of points within its boundaries; however, the estimation procedure will be the same.

Now, suppose we have two point patterns, **x** = {*x*_1_, …., *x*_*n*_**x**__} and **y** = {*y*_1_, …., *y*_*n*_**y**__, on the same observation window *W*. In order to estimate the cross *K*-function between the processes, we can proceed in the same fashion. We estimate the two intensities using the overall number of points in each pattern, ${\hat{\lambda }}_{\text{X}}=\frac{n_{\text{x}}}{\left|W\right|}$ and ${\hat{\lambda }}_{\text{Y}}=\frac{n_{\text{y}}}{\left|W\right|}$.We then restrict ourselves to *W_R_* and count neighbors between the two patterns:

$${\hat{K}}_{\text{X},\text{Y}}(r)=\frac{1}{\left|W_{R}\right|{\hat{\lambda }}_{\text{X}}{\hat{\lambda }}_{\text{Y}}}\sum_{i:x_{i}\in W_{R}}\sum_{j=1}^{n_{y}}1_{\left\{d(x_{i},y_{j})\leq r\right\}}$$

Once a simple or cross *K*-function is estimated, how do we assess whether its behavior indicates a significant spatial association, *i.e.*, a significant attraction/repulsion? In an ideal setting, we would assess whether the estimated *K* deviates significantly from quadratic growth, which is the baseline for lack of association. To detect significant deviations, we could construct “null” bands about π*r*^2^ by simulating data from Poisson random fields with intensities estimated from the data. For *K*-functions estimated on our infection marks, deviations from quadratic growth may not signify association but may indicate departures of the data from a Poisson model due to specific features of our experimental system and limits of the image analysis software. To interpret significant evidence for attraction/repulsion in terms of increased/decreased susceptibility attributable to changes in cell intrinsic properties or innate responses, we need to create statistical benchmarks that account for experimental features and limits in imaging.

Figure 1 illustrates how the underlying shape and size of the cells, which are not directly visualized, as well as the different types of stains can affect mark locations. Stains of type B, which are small and sharp, appear on membranes; therefore, marks of type B can be close to one another signifying neighboring B-infected cells. In some rare case, close B marks can also represent multiple staining on the membrane of the same B-infected cell. Stains of type A, which are larger and fuzzier, occupy the majority of a cell’s cytoplasm and some time blur into one another. It follows that cell volume exclusion constrains the minimum distance between A marks. Finally, B marks can be close to A marks signifying neighboring cells infected with the two virus strains or, much more rarely, single cells infected with both strains.

One could devise data pre-processing rules to deal with these ambiguities. Specifically, we could try to associate each A mark and each B mark to the location of a particular cell nucleus. The resulting data would be consistent with a so-called *marked* point process, *i.e.*, a set of random points in the plane (corresponding to cell locations) with random labels attached to each (the infection status). However, such rules would necessarily contain a number of arbitrary steps and would not be guaranteed to reflect the mechanisms that affect mark locations in our experimental system. Instead of pre-processing to render our data closer to ideal and then creating benchmarks based on simulations from Poisson random fields, our approach is to simulate data reproducing the mechanisms that affect mark locations, but under null scenarios that serve as benchmarks for assessing repulsion or attraction. Each of our well images consists of a circular observation window with radius 1350 in pixels (1 pixel corresponding to 6.45 microns length). Within such a window, we take nuclei, A marks, and B marks (centers of distinct stains) as observed point patterns for three processes. To assess attraction or repulsion among A marks, among B marks, and between A marks and B marks, we contrast the observed patterns with patterns representing no association simulated with the procedures described below.

### Simulating a cell support

We start with the observed nuclei marks in the well. For each mark, we independently draw a random radius from a uniform distribution between 1.5 and 2.25 pixels and create a disc centered at the mark. This results in a collection of spherical cells with diameters uniformly distributed between 19.35 and 29.025 microns (consistent with observed cell sizes). However, the more densely populated the well, the more these cells may overlap. When a newly generated cell (disc) induces one or more overlaps with existing cells (discs), we shift its center as to eliminate the overlap. The shifts do not affect the overall picture in any detectable way. Centers of simulated cells and observed nuclei marks have practically indistinguishable spatial configurations in the well. The simulated cell support we obtain with this procedure is consistent with the nuclei data and supplements it by providing spherical cell membranes and mimicking cell volume exclusion.

### Simulating A marks

Once we have the simulated cell support, we go through the cells one by one and “infect” each independently with probability $\frac{n_{A}}{n_{N}}$, where *n_N_* and *n_A_* are the number of observed nuclei and A marks, respectively. We then create simulated A marks by selecting a random location within each infected cell. This procedure is repeated to generate a total of 99 simulated A marks patterns, each with an expected number of marks equal to the observed number of A marks. These simulations lack any systematic spatial structure except for that implicitly imposed by the cell support.

### Simulating B marks

Again using the simulated cell support, we go through the cells one by one and “infect” each independently with probability $\frac{n_{B}}{n_{N}}$, where *n_B_* is the number of observed B marks. Infections with B are performed independently of infections with A (above). We then create simulated B marks by selecting a random location on the circumference of each infected cell. This procedure is repeated to generate a total of 99 simulated B marks patterns, each with an expected number of marks equal to the observed number of B marks. These simulations lack any systematic spatial structure except for that implicitly imposed by the cell support and carry no association with the simulated A marks patterns except through the underlying support.

Note that these procedures do not allow for more than one B mark on the perimeter of the same B-infected simulated cell and do allow for a simulated cell to be infected with both A and B—although with small probability. We experimented with variants in which more than one B mark could be placed on the perimeter of the same cell and/or cells were prevented from being infected with both A and B. These variants were abandoned, since they were not appreciably different in terms of benchmarking *K*-functions estimation of the data.

## 3. Results

**Figure 3:**
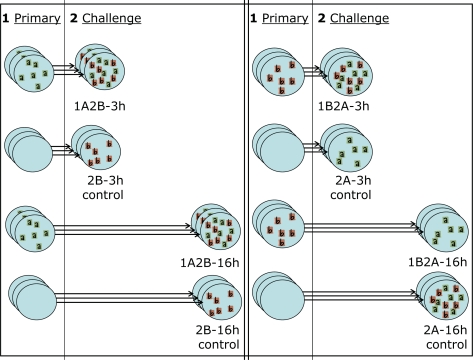
Schematic of the experimental design. Wells plated with epithelial cells are exposed to a primary infection (1), and then to a challenge (2) after a time lag of 3 hours (short arrows) or 16 hours (long arrows). On the left of the schematic, the primary infection is RSV-A and the challenge RSV-B. On the right of the schematic, the roles of the two strains are reversed. Each of these four settings has a corresponding control in which the cell culture is not exposed to the primary infection, but the challenge infection (2) is still introduced after an elapsed time of 3 or 16 hours. We therefore have a total of eight experimental settings, each of which is independently replicated three times.

As discussed in Section 1, we plate wells with epithelial cells and expose them to one strain of the virus (primary) for 1 hour. We then wash the culture and allow the infection to proceed for 3 or 16 hours (time lag). The cells are then exposed to a second strain of the virus (challenge) for 1 hour, washed, and the wells are imaged after 24 h. The experimental settings we consider here are indicated as 1A2B-3h, 1A2B-16h when the primary strain is RSV-A, the challenge is RSV-B, and the lag is 3 or 16 hours and are indicated as 1B2A-3h, 1B2A-16h when the strains used for primary and challenge infection are reversed. We also consider control settings, in which wells are exposed to the challenge after allowing an elapsed time of 3 or 16 hours from the beginning of the experiment but no primary exposure occurs. These settings are indicated as 2B-3h, 2B-16h, 2A-3h, 2A-16h. Each setting is independently replicated 3 times using separate wells. A schematic of the experimental design is provided in Figure 3, and more detail on the experimental protocols can be found in Section 5.

### Spatial association among challenge infections I: localized increase in cell susceptibility to challenge infection

**Figure 4:**
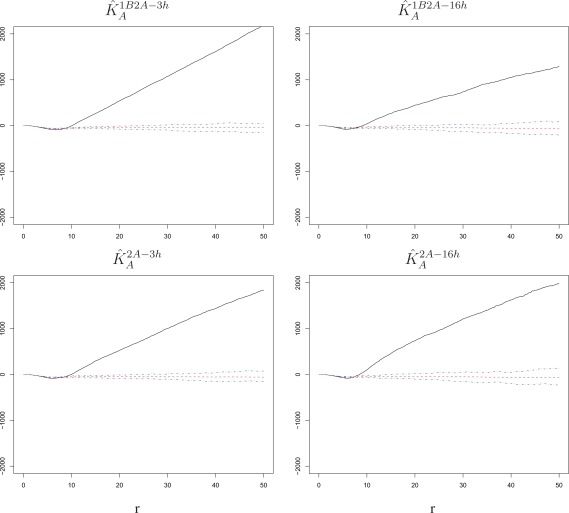
*K̂_A_* with corresponding null bands in the experimental settings 1B2A-3h, 1B2A-16h, 2A-3h and 2A-16h. Cells surrounding a cell infected by the RSV-A challenge have an increased susceptibility to the challenge itself. This is true at short and long time lags and regardless of whether the challenge was preceded by a primary infection with RSV-B. The radius *r* on the horizontal axes is measured in pixels; 1 pixel corresponds to 6.45 microns.

**Figure 5:**
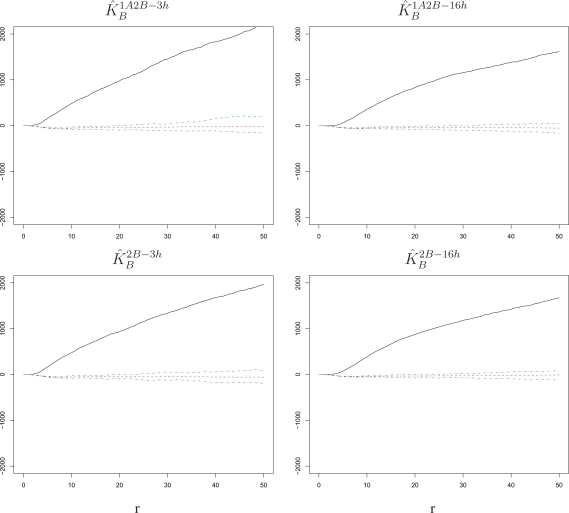
*K̂_B_* with corresponding null bands in the experimental settings 1A2B-3h, 1A2B-16h, 2B-3h, 2B-16h. Cells surrounding a cell infected by the RSV-B challenge have an increased susceptibility to the challenge itself. This is true at short and long time lags and regardless of whether the challenge was preceded by a primary infection with RSV-A. The radius *r* on the horizontal axes is measured in pixels; 1 pixel corresponds to 6.45 microns.

We start with an assessment of spatial association among marks for the challenge infection. For settings in which RSV-A is the challenge, this association is described by ${\hat{K}}_{A}^{1B2A-3h}$, ${\hat{K}}_{A}^{1B2A-16h}$, ${\hat{K}}_{A}^{2A-3h}$ and ${\hat{K}}_{A}^{2A-16h}$, which are constructed by pooling the *K*-function estimates from the three replicate wells in each setting (see Section 5 for details). The same calculations are performed for settings in which RSV-B is the challenge, producing ${\hat{K}}_{B}^{1A2B-3h}$, ${\hat{K}}_{B}^{1A2B-16h}$, ${\hat{K}}_{B}^{2B-3h}$, and ${\hat{K}}_{B}^{2B-16h}$. Pooling is also implemented for *K*-functions estimated on simulated A and B marks patterns, as to obtain “null” bands for the estimated *K*-functions (see again Section 5). Results are shown in Figures 4 and 5. In order to facilitate the visualization of departures from a quadratic growth, we subtract π*r*^2^ from the estimates *K̂* being plotted in each panel of these figures and from its null bands. Recall that the *K*-function of a Poisson random field is π*r*^2^.

In all settings considered, we detect a significant and sizable attraction (*K̂* above the bands) beyond the ranges at which cell volume exclusion may create a repulsion. Although hardly visible on the vertical scale of the figures, small negative dips do occur in the estimated *K*-functions at small *r* ranges, but they are mimicked by the simulations and hence within our bands. Thus, there is a localized increase of susceptibility of cells to the challenge virus; if one cell in a region becomes infected, there is an increased probability that cells in its proximity will also be infected. Because this occurs synchronously at the time of challenge and appears to be independent of exposure to a primary infection, we interpret it as an *intrinsic cell effect* as opposed to an innate response.

### Spatial association among challenge infections II: the role of virus strain and time lag from primary infection

**Figure 6:**
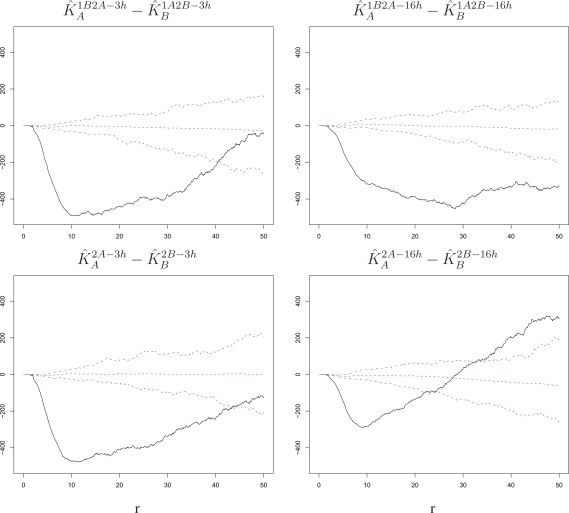
Differences of estimated *K*-functions between challenge strains with corresponding null bands. The increase in susceptibility to the challenge infection around cells infected with the challenge itself is stronger when the challenge strain is RSV-B than when it is RSV-A. This is true at short and long time lags and regardless of whether the challenge was preceded by a primary infection with the other virus strain. The radius *r* on the horizontal axes is measured in pixels; 1 pixel corresponds to 6.45 microns.

We compare estimated *K*-functions across settings to determine if the susceptible phenotype varies for the two virus strains and with the time lag between primary infection and challenge. First, we consider the differences ${\hat{K}}_{A}^{1B2A-3h}-{\hat{K}}_{B}^{1A2B-3h}$, ${\hat{K}}_{A}^{1B2A-16h}-{\hat{K}}_{B}^{1A2B-16h}$, ${\hat{K}}_{A}^{2A-3h}-{\hat{K}}_{B}^{2B-3h}$, and ${\hat{K}}_{A}^{2A-16h}-{\hat{K}}_{B}^{2B-16h}$, which represent comparisons between the two virus strains. We build null bands for them based on our simulations (see Section 5 for details on how bands for differences are derived). As shown in Figure 6, these are all sizable and significantly negative (below the bands) for a range of radii. Thus, the attraction among challenge infections is stronger for an RSV-B challenge than for an RSV-A challenge. This is true at both short and long lags and regardless of whether or not there was exposure to a primary infection, suggesting that intrinsic cell properties increase susceptibility to RSV-B more than they increase susceptibility to RSV-A infections.

**Figure 7:**
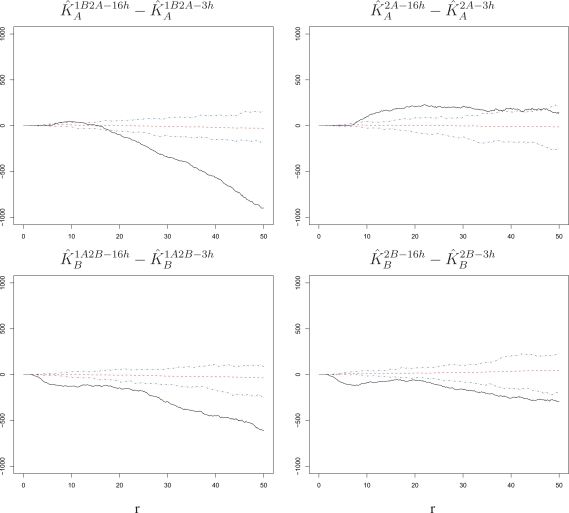
Differences of estimated *K*-functions between lags with corresponding null bands. The increase in susceptibility to the challenge infection around cells infected with the challenge itself is stronger at short than at long time lags when the challenge is preceded by a primary infection. However, the difference between short and long time lags is mostly non-significant when the challenge is not preceded by a primary infection. This is true for either order of the two strains. The radius *r* on the horizontal axes is measured in pixels; 1 pixel corresponds to 6.45 microns.

Second, we consider the differences ${\hat{K}}_{A}^{1B2A-16h}-{\hat{K}}_{A}^{1B2A-3h}$, ${\hat{K}}_{B}^{1A2B-16h}-{\hat{K}}_{B}^{1A2B-3h}$, ${\hat{K}}_{A}^{2A-16h}-{\hat{K}}_{A}^{2A-3h}$, and ${\hat{K}}_{B}^{2B-16h}-{\hat{K}}_{B}^{2B-3h}$, which represent comparisons between time lags, with the corresponding null bands. As shown in Figure 7, these differences are sizably and significantly negative (below the bands, left panels) in the presence of a primary infection. In contrast, the differences are generally non-significant (within or hardly outside the bands, right panels) when comparing time lags without a primary infection. Thus, time does not modulate attraction among challenge infections for either RSV-A or RSV-B in the absence of a primary infection. However, there is a stronger attraction among cells infected by the challenge virus following a primary infection at short time lags than at long time lags. Because this decline with time manifests itself only in the presence of a primary exposure, it provides indirect evidence of an *innate immune response* acting to limit susceptibility conferred by cell intrinsic factors.

### Spatial association among challenge infections: summary

An analysis of spatial association among challenge infections suggests that intrinsic properties render cells in close proximity to be more susceptible to infection within the time span allowed for the challenge. This intrinsic cell effect is detectable regardless of what virus strain is used in the challenge, the length of the time lag, and the presence of a primary infection (Figure 4 and Figure 5). Comparing our experimental settings, we observe stronger attraction among infected cells for RSV-B than for RSV-A, which suggests that the two strains may have different infection requirements in BEAS-2B cells (Figure 6). Moreover, the attraction tapers off with increasing time lag when the cells experience a primary infection, which suggests a possible dynamic interplay of cells responding to innate immune signals to the primary exposure and intrinsic cell properties at the time of challenge (Figure 7).

### Spatial association between primary and challenge infections I: innate response to primary infection does not overcome localized increase in cell susceptibility to challenge infection

**Figure 8:**
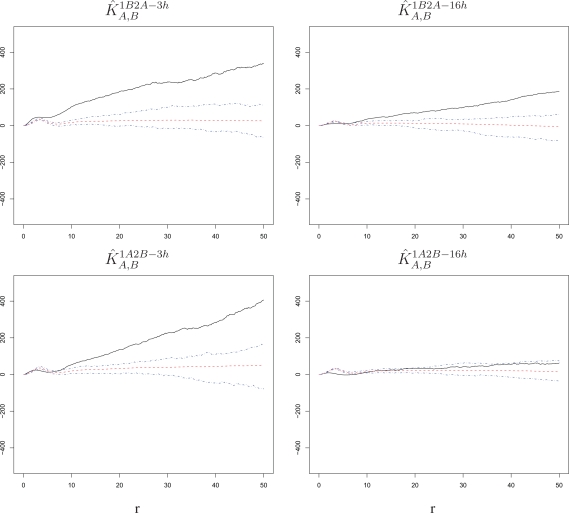
*K̂_A,B_* with corresponding null bands in the experimental settings 1B2A-3h, 1B2A-16h, 1A2B-3h, and 1A2B-16h. Cells surrounding a cell affected by the primary infection have a somewhat increased susceptibility to the challenge infection, but the effect is much weaker than the one detected among the challenge infections themselves (see Figures 4 and 5). The radius *r* on the horizontal axes is measured in pixels; 1 pixel corresponds to 6.45 microns.

Next, we seek direct evidence of an innate response effect by assessing the spatial association between cells infected with the primary virus and cells infected with the challenge virus. This association is described by the estimated cross *K*-functions ${\hat{K}}_{A,B}^{1B2A-3h}$, ${\hat{K}}_{A,B}^{1B2A-16h}$, ${\hat{K}}_{A,B}^{1A2B-3h}$, and ${\hat{K}}_{A,B}^{1B2A-16h}$. Note that we again perform pooling across replicates. Recall the cross *K*-functions are symmetric in terms of the order of the processes, and cross *K*-functions cannot be computed in control settings because those comprise only one infection. Results are shown in Figure 8; we again subtract π*r*^2^ from the pooled *K̂* being plotted in each panel and its null bands.

In three out of four settings where we have considered varying lag and order of the virus strains, we detect a significant but modest attraction (*K̂* above the bands) beyond the ranges at which double infections of the same cell may create an attraction. Small positive spikes do occur in the estimated cross *K*-functions at small *r* ranges, but they are mimicked by the simulations and are within our bands. In the fourth setting (1A2B-16h), *K̂* is within the bands. Thus, cells surrounding a cell infected with the primary virus have a somewhat increased susceptibility to infection with the challenge virus. By and large, the effect detected here is much smaller than the intrinsic cell effect discussed above. We interpret this as evidence that cells do not mount innate responses strong enough to render them measurably less susceptible to a challenge infection over the time scales of our study. However, the modest increase in susceptibility to the challenge for cells near a primary infected cell may indeed be due to an innate immune mediated damping of the intrinsic cell phenotype, which increases local susceptibility of cells to infection.

### Spatial association between primary and challenge infections II: the role of virus order and time lag from primary infection

**Figure 9:**
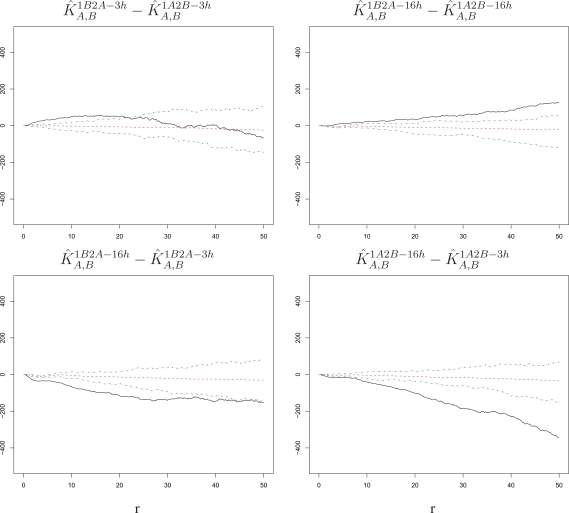
Differences of estimated cross *K*-functions between virus strain orders and between lags with corresponding null bands. The increase in susceptibility to the challenge infection around cells infected with the primary infection does not differ significantly depending on the order of the virus strains. In other words, it does not matter which is used as primary infection and which is used as challenge. However, the effect is somewhat stronger at short than at long time lags. The radius *r* on the horizontal axes is measured in pixels; 1 pixel corresponds to 6.45 microns.

Comparing estimated cross *K*-functions across settings in Figure 9, we find that the order of the virus strains, represented by the differences ${\hat{K}}_{A,B}^{1B2A-3h}-{\hat{K}}_{A,B}^{1A2B-3h}$ and ${\hat{K}}_{A,B}^{1B2A-16h}-{\hat{K}}_{A,B}^{1A2B-16h}$, is non-significant at either 3 or 16 hour time lag (curves within or hardly outside the bands). However, there is an effect of time lag based on the differences ${\hat{K}}_{A,B}^{1B2A-16h}-{\hat{K}}_{A,B}^{1B2A-3h}$ and ${\hat{K}}_{A,B}^{1A2B-16h}-{\hat{K}}_{A,B}^{1A2B-3h}$, which are significantly, though modestly negative. Thus, the association between cells infected with the primary and challenge strains is somewhat stronger at short than at long time lags, regardless of the order of the virus strains. This observation is consistent with the generation of an innate immune response to the primary infection, which develops over the 16 hour time course and counteracts the increased susceptibility conferred by cell intrinsic factors.

### Spatial association between primary and challenge infections: summary

An analysis of spatial association between infections with primary and challenge viruses suggests that cells do not mount innate responses strong enough to measurably decrease their susceptibility (cause a repulsion in our analysis) to a challenge infection over the time scales of our study. However, cells do appear to initiate an innate response that tends to decrease neighboring cell susceptibility over time. This effect is partially masked by the intrinsic cell factors that enhance local cell susceptibility to infection.

## 4. Conclusions

In this article, we used methods from spatial statistics to explore how a primary RSV infection alters the susceptibility or resistance of cells to a challenge with a heterologous RSV strain. Using these methods allowed us to characterize infections in terms of their locations and proximity to one another and to study their spatial association in the cultures (attraction or repulsion; *i.e.*, a tendency for infected cells to be closer or more distant than expected by chance) under different experimental settings. In turn, this allowed us to investigate how susceptibility to infection is affected by local conditions such as intrinsic cell properties associated with cell cycle or maturation state or by signaling among nearby cells responding to viral infection through the innate immune system.

Spatial associations detected among challenge infections provided a clear finding: intrinsic cell properties appear to render cells neighboring an infected cell substantially more susceptible to infection. This leads to an attraction among cells infected by the challenge virus independent of the presence of a primary infection. Of interest, this effect appears more marked for RSV-B than for RSV-A infections, suggesting a possible difference between the two strains in the infection dynamics.

Over the experimental time frame of our study, the innate immune response is harder to characterize because its effects are partially masked by the effects of intrinsic cell features. Spatial associations detected between primary and challenge infections do not indicate a decrease in susceptibility at either of the time lags we considered. When the culture is exposed to a challenge after a short lag, the cells have not had enough time to mount strong innate responses. Consequently, the increased susceptibility due to the intrinsic cell effect acts with little opposing forces around primary infections and the net result is an overall increased susceptibility of cells to the challenge virus. However, when the culture is exposed to a challenge after a longer time lag, innate responses are better developed. With the intrinsic cell effect now somewhat counteracted by innate immune responses, the net result is a still evident but milder increase in susceptibility of cells to the challenge. Our experimental system was designed to prevent the possibility of cell-to-cell viral spread. The time line we used captures a single cycle of infection, with susceptibility of cells to the challenge assessed prior to the release of infectious virus by the primary infected cells (see Methods). Moreover, susceptibility to the challenge is determined by exposing cells to a second inoculum and can be discriminated from the primary infection based on staining characteristics.

Research on virus interactions with cell innate factors typically does not exploit the important information available from the spatial arrangement of infected cells. As single cell-based assays and primary cultures become more widely used to study short term responses of cells to viral infections, spatial statistics methods such as those used in this study will be valuable to unravel local variation in these responses. In our case, we found that susceptibility to infection was variable even though cells should be synchronized based on plating and passage history. This difference in cell susceptibility to RSV infection could be associated with receptors or intracellular factors needed in the viral life cycle that are differentially expressed in actively replicating cells and those that are resting. The underlying mechanism may be important because it enhances susceptibility to RSV-B infection compared to RSV-A.

From a technical point of view, our analysis revolved around spatial point processes, *K*-functions, standard methodology to estimate such functions, and simulation procedures we designed to detect significant spatial association accounting for complicating issues specific to our data. These include features of our experimental cultures, the nature of the stains used to visualize nuclei and infections, and limits in the image analysis software which render the two dimensional mark coordinates at out disposal imperfect proxies for the locations of cells and their infection status. Importantly, our estimation of the *K*-functions and significance assessment of spatial associations were greatly simplified by the assumption that nuclei and infections marks are realizations of *homogeneous* point processes. However, this is not an obvious assumption. Depending on plating protocols and other factors, tissue cultures can and often do present inhomogeneous cell distributions. Following experience accrued in previous runs of the same experimental system we were able to improve our protocols as to obtain wells with fairly homogeneous cell distributions. Because the cell support is homogeneous and the MOIs are high, we also obtain fairly homogeneous distributions for the infections (see Section 5). However, when cells, and consequently infections, present strong patterns in their spatial distributions, marks may need to be modeled as inhomogeneous point processes, and *K*-function estimation and significance assessment become more complex [11–14]. Preliminary data was collected using different MOIs with lower values for the primary infections and higher values for the challenge infections. This preliminary data was consistent with the results shown in the paper; however lower MOIs resulted in sparser point patterns making the data less reliable for statistical analysis.

The results from this article and [15] suggest that further progress in understanding infections and co-infections through *in vitro* studies will rely crucially on developing appropriate spatio-temporal models for the data they produce. In [15], the authors construct a dynamic model for the susceptibility of cells in the same experimental setup as presented here. Spatial structure in not explicitly considered in [15], and the diffusion of the virus through the culture is modeled “in bulk” with specific biological mechanisms that predict the aggregate infection counts within a culture. Conversely, our study models spatial structure but not temporal dynamics. We are currently engaged in an effort to combine these approaches through models that can include explicit mechanisms for cell susceptibility through time and space. It should be noted that developing spatio-temporal models for the type of experimental setup used here and in [15] poses a significant challenge. In particular, if we cannot observe a single cell culture at multiple time points, our observations will be independent through time limiting the range of methods at our disposal.

## 5. Methods

### Experimental protocols

The human bronchial epithelial cell line (BEAS-2B) was purchased from American Type Culture Collection (ATCC, Manassas, VA) and was maintained in serum-free growth medium (LHC-8; Invitrogen, Carlsbad, CA). All experiments were conducted with cells in their sixth passage in order to minimize heterogeneity that can arise with different culture history. HEp-2 cells were maintained in OptiMEM (Invitrogen, Carlsbad, CA) supplemented with 2% fetal calf serum (FCS; HyClone Laboratories, Salt Lake City, UT), 100 IU/mL penicillin and 100ug/mL streptomycin and *β*-mercaptoethanol. Human respiratory syncytial virus B (ATCC) and recombinant RSVA2eGFP (a gift from Dr. M. Teng) were propagated in HEp-2 cells as described in (Gias et al., 2008; Mbiguino and Menezes, 1991). HEp-2 supernatants containing infectious RSV were collected and the virus was precipitated using a final concentration of 10% polyethylene glycol (Sigma, St. Louis, MO). The precipitate was dissolved in NT (50 mM Tris-HCl, 150 mM NaCl, pH 7.5) buffer, and overlaid on a discontinuous 60%, 45% and 30% sucrose gradient made up in NT buffer. After centrifugation for 100 min at 112,000 g in a SW28 rotor, the virus was collected from the 30–45 % interface. The virus was stored in small aliquots at −80°C until use.

BEAS-2B cells were seeded in 1 × 10^5^ cells per well in 24-well plate and incubated for 20 hourse at 37°C, then exposed to RSVB (0.5MOI) for 1 hour. Cells were washed to remove unattached virus and media was replaced. At 3 and 16 hours post-infection, BEAS-2B cells were secondarily challenged with RSVA2eGFP at an MOI of 0.5 for 1 hour and washed. The experiment was also performed reversing the order of infection. Twenty four hours post secondary RSV infection, BEAS-2B cells were washed with phosphate-buffered saline (PBS) and fixed with 4% paraformaldehyde. Then, cells were permeabilized and blocked in PBS containing 1% BSA and 0.3% Triton X-100 for 1h at room temperature. For RSVB staining, cells were incubated with anti-RSVB monoclonal antibody (MAB8582, Chemicon, Billerrica, MA) followed by incubation with Alexa fluor 568 goat anti-mouse (Invitrogen, Carlsbad, CA) and stained with 300nM of DAPI. Green, red and blue images were captured by fluorescence microscope and analyzed using Image Pro Plus software version 6.3 [16].

### Image analysis

The image analysis software package [16] was used to produce stain sizes and 2D spatial coordinates for nuclei, RSV-A, and RSV-B marks. Nuclei were handled by setting the intensity range selection to 0, 80, 255. For RSV-A stains, we used the following software options: intensity range selection 0, 37, 255 followed by auto-split and watershed-split options. For RSV-B stains we used the following software options: contrast enhancement of 44, 50, 1.9, followed by sharpen filter 3×3 1 Pass, followed by median filter 3×3 1 Pass, and intensity range selection of 0, 42, 255. To ensure consistency and data reliability, we created scripts that automated the same procedures and software options/parameter settings for analysis of all well images.

### Pooled estimates of K-functions

The results in Section 3 were presented using estimates of *K*-functions and cross *K*-functions pooled across the three replicates available for each experimental setting. Here we provide details on the pooling procedure. Let ℓ = 1, 2, 3 index replicates for the same experimental setting. Each replicate produces a point pattern **x**_(ℓ)_ = {*x*_(ℓ)1_, …, *x*_(ℓ)*n*_(ℓ)__} on an observation window *W*^(ℓ)^ of the same shape and size (a circle with radius 1350 pixels), and in all three cases, the buffered subregion $W_{R}^{\left(\ell \right)}\subset W^{\left(\ell \right)}$ has the same shape and size (a circle with radius 1300 pixels). We obtain three estimates of the *K*-function using the formula introduced in Section 2

$${\hat{K}}_{\left(\ell \right)}(r)=\frac{1}{\left|W_{R}^{\left(\ell \right)}\right|{\hat{\lambda }}_{\left(\ell \right)}^{2}}\sum_{i:x_{(\ell )i}\in W_{R}^{\left(\ell \right)}}\sum_{j\neq i}1_{\left\{d(x_{(\ell )i},x_{(\ell )j})\leq r\right\}}$$

for ℓ = 1, 2, 3, and we form a pooled estimate taking a weighted average of the three

$$\begin{array}{l} \hat{K}(r)=\sum_{\ell =1}^{3}\frac{\left|W_{R}^{\left(\ell \right)}\right|}{\left|W_{R}^{\left(1\right)}\right|+\left|W_{R}^{\left(2\right)}\right|+\left|W_{R}^{\left(3\right)}\right|}{\hat{K}}_{\left(\ell \right)}(r)\\ \;\;=\sum_{\ell =1}^{3}\frac{\left|W_{R}^{\left(\ell \right)}\right|}{\left|W_{R}^{\left(1\right)}\right|+\left|W_{R}^{\left(2\right)}\right|+\left|W_{R}^{\left(3\right)}\right|}\left[\frac{1}{\left|W_{R}^{\left(\ell \right)}\right|{\hat{\lambda }}_{\left(\ell \right)}^{2}}\sum_{i:x_{(\ell )i}\in W_{R}^{\left(\ell \right)}}\sum_{j\neq i}1_{\left\{d(x_{(\ell )i},x_{(\ell )j})\leq r\right\}}\right] \end{array}$$

in which each of the estimates, *K̂*_(ℓ)_, contributes in proportion to the size of its $W_{R}^{\left(\ell \right)}$ and ${\hat{\lambda }}_{\left(\ell \right)}=\frac{n_{\left(\ell \right)}}{{\left|W\right|}^{\left(\ell \right)}}$, ℓ = 1, 2, 3 [11]. Note though that since the three subregions have the same size in our data, the above corresponds to taking a simple averaging of the three estimates, *K̂*_(ℓ)_. The same procedure is used to produce pooled estimates of cross *K*-functions. Other approaches to pooling for *K*-function estimation exist, such as using weights based on the cardinality of each point pattern [17–19]. However, these result in similar estimates from our data, because the number of points, *n*_(ℓ)_, are similar across replicates of the same experimental setting.

### Null bands for pooled estimates of K-functions

To produce the null band for a pooled estimate, *K̂*, (Figures 4, 5 and 8) we also perform pooling across the 99 simulated point patterns generated for each of the three replicates. When we estimate a *K*-function, we do so on a discrete grid of 500 *r* values evenly spaced in the interval (0, 50). The resulting numbers, which are obtained from the point pattern observed in a replicate and the 99 point patterns simulated for that replicate, are arranged in an array $M_{500\times 100}^{\left(\ell \right)}$. The three arrays (one for each replicate) are then averaged to produce $M_{500\times 100}=\frac{1}{3}\left[\sum_{\ell =1}^{3}M_{500\times 100}^{\left(\ell \right)}\right]$. For each row of the array *M*_500×100_, we compute the minimum, the mean, and the maximum of the 99 columns corresponding to simulations (*i.e.*, columns 2 through 100). The probability that the pooled estimate of the *K*-function is below (above) the minimum (maximum) under the null hypothesis of no spatial association is

$$P\left(\hat{K}(r)<\underset{j=2,\ldots ,100}{\text{min}}M_{500\times 100}[r,j]\right)=P\left(\hat{K}(r)>\underset{j=2,\ldots ,100}{\text{max}}M_{500\times 100}[r,j]\right)\leq \frac{1}{99+1}$$

[11]. For every *r* in our grid, the minimum and the maximum provide 1%-lower and 99%-upper limits. The null band is constructed from 500 such lower and upper limits. It should be stressed that since we are essentially performing 500 hypothesis tests based on the same simulated data, such a null band does not control the overall level at 2%. The same procedure is used to produce null bands for pooled estimates of cross *K*-functions.

### Null bands for differences between pooled estimates of K-functions

Here we describe how the null band is constructed when considering the difference between two pooled estimates of *K*-functions (Figures 6, 7 and 9). We start with the arrays $M_{500\times 100}^{\left(\ell ,s\right)}$ produced for each of the three replicates ℓ = 1, 2, 3 of two experimental settings *s* = 1, 2. We then form difference arrays $D_{500\times 100}^{\left(\ell \right)}=M_{500\times 100}^{\left(\ell ,2\right)}-M_{500\times 100}^{\left(\ell ,1\right)}$, ℓ = 1, 2, 3, and we average them to obtain $D_{500\times 100}=\frac{1}{3}\sum_{\ell =1}^{3}D_{500\times 100}^{\left(\ell \right)}$. For each row of *D*_500×100_, we compute the minimum, the mean, and the maximum of the 99 columns corresponding to simulations (*i.e.*, columns 2 through 100). The minimum and maximum across each of the 500 rows provide lower and upper limits under the null hypothesis of no spatial association for the difference in the estimates *K̂* at a given *r*. The null band for the differences is constructed from these 500 lower and upper limits but does not control the overall level at 2%.

### Tests for homogeneity

To verify that our data meets the homogeneity assumption, we started by inspecting the A marks and B marks in our well images. We estimated *inhomogeneous* intensities, which showed no systematic placement of the A marks and B marks in particular regions of the wells. With this preliminary evidence, we further assumed that if the nuclei are homogeneous then there is no reason to expect the A marks and B marks to present strong inhomogeneity. We proceeded to test for nuclei homogeneity in each well. In order to do so, we benchmarked the observed nuclei point patterns against a homogeneous Poisson random field. Note that we know that the nuclei cannot be modeled by a Poisson field due to the natural cell exclusion mechanism of the cells. However, the testing procedure we employ divides the observation window into a grid of squares *Q_i_, i* = 1*, …, k* with sides of 159 pixels. Note also that only full sized squares inside the window are used. The departures from a Poisson field are at small scales compared to the size of these squares. Our null hypothesis is that *H_o_* : *The nuclei point pattern within a well is a realization of a homogeneous point process*, and the alternative is that *H_a_ : The nuclei point pattern within a well is not a realization of a homogeneous point process*. Due to the properties of the homogeneous Poisson random field, we know that, under the null hypothesis, (i) the number of points in each of the squares *N*(*Q_i_*) is distributed as a Poisson random variable with mean λ × *|Q_i_|* and (ii) the number of points in each of the squares is independent from the number of points in the other squares. Thus we can consider the test statistic $\chi^{2}=\sum_{i=1}^{k}{\left(\frac{N(Q_{i})-\hat{\lambda }\times \left|Q_{i}\right|}{\sqrt{\hat{\lambda }\times \left|Q_{i}\right|}}\right)}^{2}$ which, under the null, is approximately distributed as a chi-squared distribution with *k* − 1 degrees of freedom. We performed 24 such tests, one for each well under consideration, and failed to reject the null at level 5% for 17 out of 24 cases. Using graphical diagnostic devices we were also able to verify that, in all cases, the counts of nuclei within squares (*N*(*Q_i_*)) were by and large consistent with a Poisson distribution except for a few extreme values—*i.e.*, some very low and very high counts. The tests were thus performed after “trimming” out the 5% bottom and top counts in each well. Notably, overall visual consistency with Poisson counts holds even in the 7 wells where a nominally significant departure from homogeneity was detected by the testing procedure. In all, we consider this enough evidence to rely on homogeneity in our formulation and estimation of *K*-functions. [20–22]
